# A Fine Balance of Synaptophysin Levels Underlies Efficient Retrieval of Synaptobrevin II to Synaptic Vesicles

**DOI:** 10.1371/journal.pone.0149457

**Published:** 2016-02-12

**Authors:** Sarah L. Gordon, Callista B. Harper, Karen J. Smillie, Michael A. Cousin

**Affiliations:** 1 Florey Institute of Neuroscience and Mental Health, The University of Melbourne, 30 Royal Parade, Parkville, 3052, Victoria, Australia; 2 Centre for Integrative Physiology, George Square, University of Edinburgh, EH8 9XD Edinburgh, United Kingdom; UPR 3212 CNRS -Université de Strasbourg, FRANCE

## Abstract

Synaptobrevin II (sybII) is a vesicular soluble NSF attachment protein receptor (SNARE) protein that is essential for neurotransmitter release, and thus its correct trafficking to synaptic vesicles (SVs) is critical to render them fusion competent. The SV protein synaptophysin binds to sybII and facilitates its retrieval to SVs during endocytosis. Synaptophysin and sybII are the two most abundant proteins on SVs, being present in a 1:2 ratio. Synaptophysin and sybII are proposed to form a large multimeric complex, and the copy number of the proteins in this complex is also in a 1:2 ratio. We investigated the importance of this ratio between these proteins for the localisation and trafficking of sybII in central neurons. SybII was overexpressed in mouse hippocampal neurons at either 1.6 or 2.15–2.35-fold over endogenous protein levels, in the absence or presence of varying levels of synaptophysin. In the absence of exogenous synaptophysin, exogenous sybII was dispersed along the axon, trapped on the plasma membrane and retrieved slowly during endocytosis. Co-expression of exogenous synaptophysin rescued all of these defects. Importantly, the expression of synaptophysin at nerve terminals in a 1:2 ratio with sybII was sufficient to fully rescue normal sybII trafficking. These results demonstrate that the balance between synaptophysin and sybII levels is critical for the correct targeting of sybII to SVs and suggests that small alterations in synaptophysin levels might affect the localisation of sybII and subsequent presynaptic performance.

## Introduction

Neuronal activity relies on the efficient reformation of fusion-competent synaptic vesicles (SVs) to sustain neurotransmitter release. This requires the packaging of vesicular membrane with the correct complement of SV cargo proteins at a defined stoichiometry [1,2]. It is becoming increasingly evident that protein-loading of SVs during endocytosis is a highly regulated process, requiring classical clathrin adaptor proteins, monomeric adaptor proteins, and specialised intrinsic vesicle proteins that traffic essential vesicular cargo [3,4].

Synaptobrevin II (sybII) is the most abundant SV protein, with approximately 70 copies per SV [1], and is a vesicular soluble NSF attachment protein receptor (v-SNARE). SybII confers fusion competence to SVs and drives exocytosis through the formation of SNARE complexes with the plasma membrane target SNAREs, syntaxin and SNAP-25 (synaptosomal-associated protein, 25kDa) [5,6]. SybII is an essential SV cargo protein, since its absence from SVs results in the cessation of evoked neurotransmitter release and early postnatal death [7].

Synaptophysin is the second-most abundant SV protein, with approximately 32 copies per SV. It interacts directly with sybII, most likely through their transmembrane domains [8–11]. Synaptophysin is essential for the efficient retrieval of sybII to SVs during endocytosis; the absence of synaptophysin results in the mislocalisation of sybII from the nerve terminal and its stranding at the plasma membrane due to defective retrieval during compensatory endocytosis [12,13].

The synaptophysin-sybII complex is proposed to be a large multimeric structure, with synaptophysin forming a central hexameric core that binds six sybII dimers, and thus the stoichiometry of synaptophysin to sybII in this complex is 1:2 (6 synaptophysin molecules bind to 12 sybII molecules) [11,14]. This closely matches the stoichiometry of the two proteins on SVs, with sybII being approximately 2-fold more abundant than synaptophysin [1,2]. Together, this suggests that the fine balance between synaptophysin and sybII levels may be key to the correct targeting of sybII to SVs.

To investigate the importance of synaptophysin levels in sybII localisation, we overexpressed sybII at differing levels in primary cultures of wild-type mouse hippocampal neurons in the absence or presence of different concentrations of exogenous synaptophysin. We show that in the absence of exogenous synaptophysin, overexpressed sybII displays defective targeting to nerve terminals, an increased plasma membrane localisation and slow retrieval during SV endocytosis. Correct targeting and trafficking of sybII is rescued by introducing exogenous synaptophysin to these neurons in an approximate 1:2 ratio, demonstrating that synaptophysin and sybII must be present in their correct stoichiometry to ensure accurate trafficking of sybII.

## Materials and Methods

### Materials

SybII-pHluorin was provided by Prof. G. Miesenbock (Oxford University, UK). Synaptophysin-mCerulean (syp-mCer) was generated as outlined [12]. Neurobasal media, B-27 supplement, penicillin/streptomycin, Minimal Essential Medium (MEM), and Lipofectamine 2000 were obtained from Invitrogen (Paisley, UK). Rabbit polyclonal anti-synaptophysin (catalogue number 101002) antibody was from Synaptic Systems (Goettingen, Germany). Chicken polyclonal anti-GFP (ab13970) and rabbit polyclonal anti-VAMP2 (anti-sybII) (ab3347) primary antibodies were from Abcam (Cambridge, UK). Secondary Alexa468 goat anti-chicken (A11039) and Alexa568 polyclonal goat anti-rabbit (A21069) were from ThermoFisher Scientific (Loughborough, UK). All other reagents were obtained from Sigma-Aldrich (Poole, UK).

### Methods

#### Hippocampal neuronal cultures

All animal work was performed in accordance with the UK Animal (Scientific Procedures) Act 1986, under Project and Personal Licence authority and was approved by the Animal W6aelfare and Ethical Review Body at the University of Edinburgh. Specifically, all animals were killed by schedule 1 procedures (decapitation for prenatal and cervical dislocation for postnatal) in accordance with UK Home Office Guidelines. Mouse colonies were housed in standard open top caging on a 14 hour light / dark cycle (light 07:00–21:00). Breeders were fed RM1 chow, whereas mice were maintained on RM3 chow. Dissociated primary hippocampal enriched neuronal cultures were prepared from E17.5 C57BL/6J mouse embryos of both sexes as outlined [15]. Single-cell suspension of hippocampal neurons were plated at a density of 3–5 x10^4^ cells/coverslip on poly-D-lysine and laminin-coated 25 mm coverslips. Cells were transfected after 7–8 days in culture with Lipofectamine 2000 as described [12]. In all experiments 2 constructs were coexpressed: sybII-pHluorin (1 or 2 μg) was cotransfected with either mCerulean empty vector (mCer) (1 or 2 μg) or syp-mCer (1 or 2 μg). Cells were fixed or imaged after 13–16 days in culture.

#### Immunolabelling

Immunolabelling of transfected cultured hippocampal neurons was performed as described [12]. Neurons were co-immunolabelled with anti-GFP (1: 5000) to identify transfected neurons, and either anti-synaptophysin (1: 500) or anti-sybII/VAMP2 (1: 1000) to determine protein expression levels. Secondary antibodies were used at 1: 500. GFP immunolabelling was visualised at 480 nm excitation (with emission monitored using a 526/26 nm band-pass filter and 495 nm dichroic filter), whereas sybII or synaptophysin immunolabelling was visualised at 550 nm excitation (with emission monitored using a long-pass >565 nm filter and a 560 dichroic filter). Identically sized regions of interest were placed over transfected puncta and non-transfected puncta in the same field of view, along with background regions. The level of overexpression was calculated by subtracting background autofluorescence prior to ratioing transfected/non-transfected synaptophysin or sybII expression levels.

#### Fluorescent imaging protocols

Hippocampal cultures were mounted in a Warner imaging chamber with embedded parallel platinum wires (RC-21BRFS) and placed on the stage of Zeiss Axio Observer D1 epifluorescence microscope. Neurons transfected with mCer vectors were visualised with a Zeiss Plan Apochromat x40 oil immersion objective (NA 1.3) at 430 nm excitation, whereas sybII-pHluorin was visualised at 500 nm excitation. The same emission collection was applied in both instances (using a 525 nm dichroic filter and long-pass emission filter, >535 nm). Cultures were stimulated with a train of 300 action potentials delivered at 10 Hz (100 mA, 1 ms pulse width). Cultures were subjected to continuous perfusion with imaging buffer (in mM: 136 NaCl, 2.5 KCl, 2 CaCl2, 1.3 MgCl2, 10 glucose, 10 HEPES, pH 7.4 supplemented with 10 μM 6-cyano-7-nitroquinoxaline-2,3-dione and 50 μM DL-2-Amino-5-phosphonopentanoic acid), and were then challenged with alkaline imaging buffer (50 mM NH_4_Cl substituted for 50 mM NaCl) to reveal total sybII-pHluorin fluorescence. Fluorescent images were captured at 4 s intervals using a Hamamatsu Orca-ER digital camera and processed offline using Image J 1.43 software. Regions of interest of identical size were placed over nerve terminals and the total fluorescence intensity was monitored over time. Only regions that responded to action potential stimulation were selected for analysis. All statistical analyses were performed using Microsoft Excel and GraphPad Prism software. The sybII-pHluorin fluorescence change was calculated as FΔ/F0, and n refers to the number of individual coverslips examined.

Surface-localised sybII-pHluorin was revealed by bathing neurons in imaging buffer, and then perfusing with acidic imaging buffer (20 mM 2-(*N*-morpholino)ethanesulfonic acid substituted for 10 mM HEPES, pH 5.5) to quench surface sybII-pHluorin fluorescence and retain background autofluorescence. Neurons were washed in imaging buffer, and then exposed to alkaline imaging buffer to reveal total sybII-pHluorin fluorescence. The surface fraction of sybII-pHluorin as a percentage of total was calculated using the following equation: [(basal fluorescence—acidic fluorescence)/(alkaline fluorescence—acidic fluorescence)] x 100.

The diffuseness of sybII-pHluorin fluorescence along axons was determined by calculating the coefficient of variation (CV) [13,16]. Neurons were exposed to alkaline imaging buffer to reveal total sybII-pHluorin fluorescence. SybII-pHluorin axon segments (>100 pixels) were traced, and the SD/mean (CV) fluorescence intensity determined. N refers to the mean of 5 different >100 pixel axonal segments on an individual neuron on a single coverslip.

## Statistical analysis

All data analysis was performed in Microsoft Excel and GraphPad Prism 6. A one-way ANOVA with Holm-Šídák post-hoc tests were used to compare more than two groups, while a two-way ANOVA with Tukey’s multiple-comparison post-test was performed to compare multiple groups over time. In all analyses, the sample size (n) was taken to be the number of independent experiments. All data are presented as mean values ± standard error of the mean (SEM).

## Results

### Synaptophysin expression levels are critical for retention of sybII at nerve terminals

**Fig 1:**
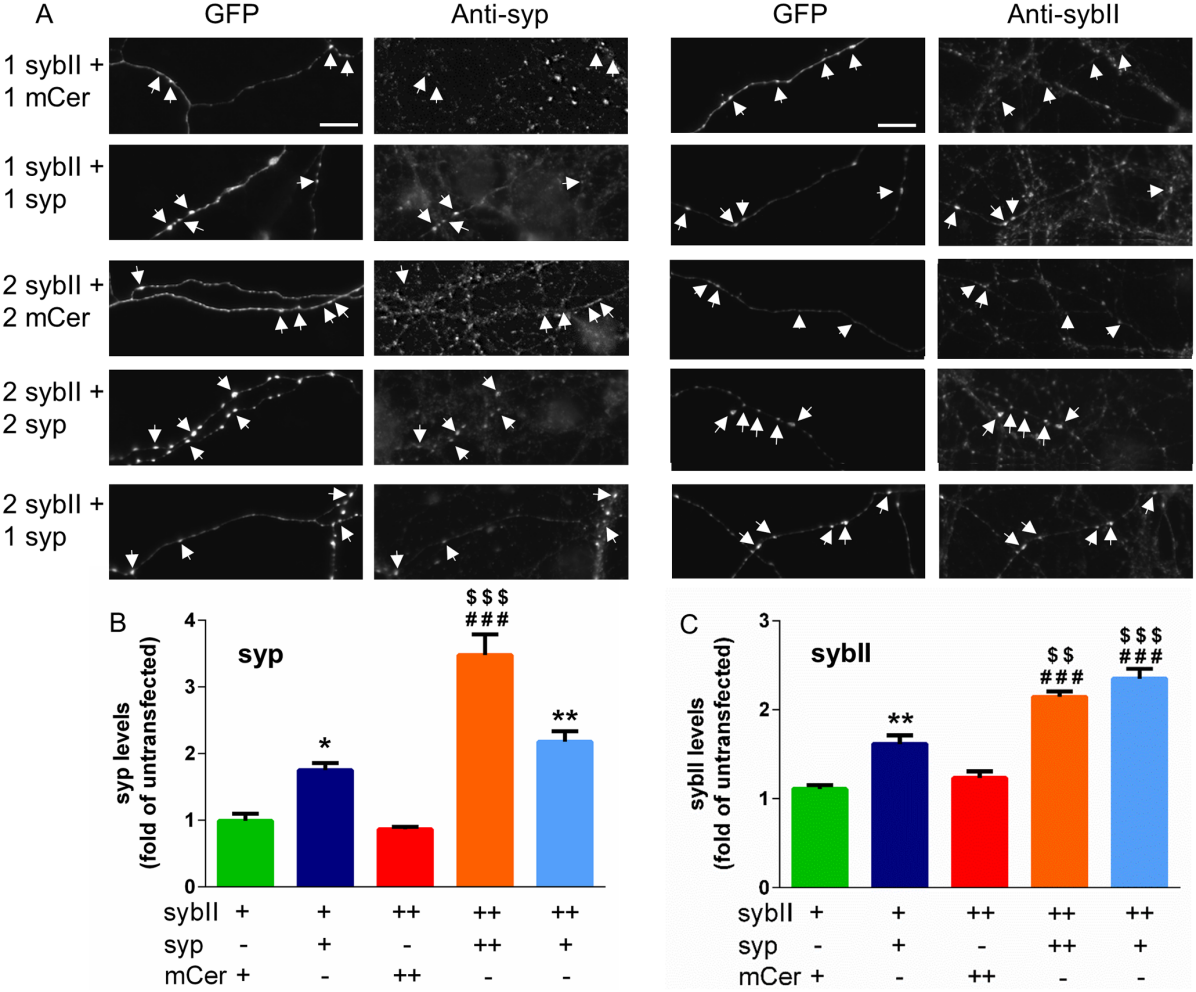
Overexpression of synaptophysin and sybII in hippocampal neurons. Hippocampal neurons were cotransfected with sybII-pHluorin (1 or 2 μg) and either mCer (1 or 2 μg) or syp-mCer (syp, 1 or 2 μg). Neurons were fixed and immunolabelled with anti-GFP and either anti-synaptophysin or anti-sybII antibodies. A: representative images of transfected neurons immunolabelled with anti-GFP (left) and either anti-synaptophysin or anti-sybII (right) antibodies. Numbers indicate μg of each construct used. Arrows indicate puncta from transfected neurons. Scale bar represents 15 μm. B, C: Level of synaptophysin (B) or sybII (C) overexpression, + indicates 1 μg of each construct used. Data displayed as mean ± SEM, n = 3 for all except 1 μg sybII-pHluorin + 1 μg syp and 2 μg sybII-pHluorin + 2 μg syp in B (n = 4). p < 0.05 (*), < 0.01 (**) against 1 μg sybII-pHluorin + 1 μg mCer; p < 0.001 (###) against 2 μg sybII-pHluorin + 2 μg mCer; p < 0.01 ($ $), 0.001 ($ $ $) against 1 μg sybII-pHluorin + 1 μg syp, one-way ANOVA with Holm-Šídák’s multiple-comparison post-test.

Synaptophysin and sybII exist in a 1:2 ratio on SVs, which is also reflected in the relative copy number of the two proteins when forming large multimeric complexes. This suggests that the relative proportion of each cargo molecule is key to normal neuronal function and that perturbation of this ratio may result in disrupted sybII trafficking. We tested this hypothesis by transfecting wild-type hippocampal neurons with increasing concentrations of exogenous sybII in the absence or presence of increasing concentrations of exogenous synaptophysin. We first quantified the expression levels of these two proteins at nerve terminals in our model system (Fig 1). Synaptophysin was overexpressed either 1.8–2.2-fold (1 μg syp-mCer) or 3.5-fold (2 μg syp-mCer) over endogenous levels at nerve terminals (Fig 1B). When increasing amounts of sybII-pHluorin (1 or 2 μg) were introduced to neurons in the absence of additional synaptophysin, no obvious increase in nerve terminal expression of sybII was observed (1.1–1.2-fold overexpression, Fig 1C). However in the presence of co-expressed synaptophysin, the extent of exogenous sybII expression in nerve terminals increased to 1.6-fold (1 μg sybII) or between 2.15 and 2.35-fold (2 μg sybII) over endogenous sybII levels (Fig 1C).

Intriguingly, 1 μg of exogenous synaptophysin was sufficient to support the nerve terminal expression of 2 μg exogenous sybII as effectively as a larger increase in synaptophysin (2 μg). To determine how this translates into their molecular ratio in nerve terminals, we scaled the known copy numbers of endogenous synaptophysin and sybII on SVs (32 and 70 respectively) to the nerve terminal expression levels of the proteins in Fig 1B and 1C. In this calculation we assumed there are approximately 300 SVs per nerve terminal [2,17], each with 32 and 70 copies of synaptophysin and sybII respectively. Therefore in the absence of any overexpression nerve terminals will have 9600 copies of synaptophysin and 21000 copies of sybII, in close agreement with previous estimates [2]. We calculated that 1 μg of DNA encoding exogenous synaptophysin and 2 μg DNA encoding exogenous sybII results in 21000 and 49500 relative copy numbers respectively in terms of the total nerve terminal expression level of these two proteins, which equates to an approximately 1:2 ratio (1: 2.36). Furthermore, 2 μg of exogenous synaptophysin with 2 μg of exogenous sybII equated to an approximately 1:1 ratio (1: 1.34). Together, this suggests that the presence of exogenous synaptophysin is required to support an increase in sybII localisation at nerve terminals, and that a putative 1:2 ratio of synaptophysin: sybII is sufficient to fully support retention of sybII within nerve terminals.

**Fig 2:**
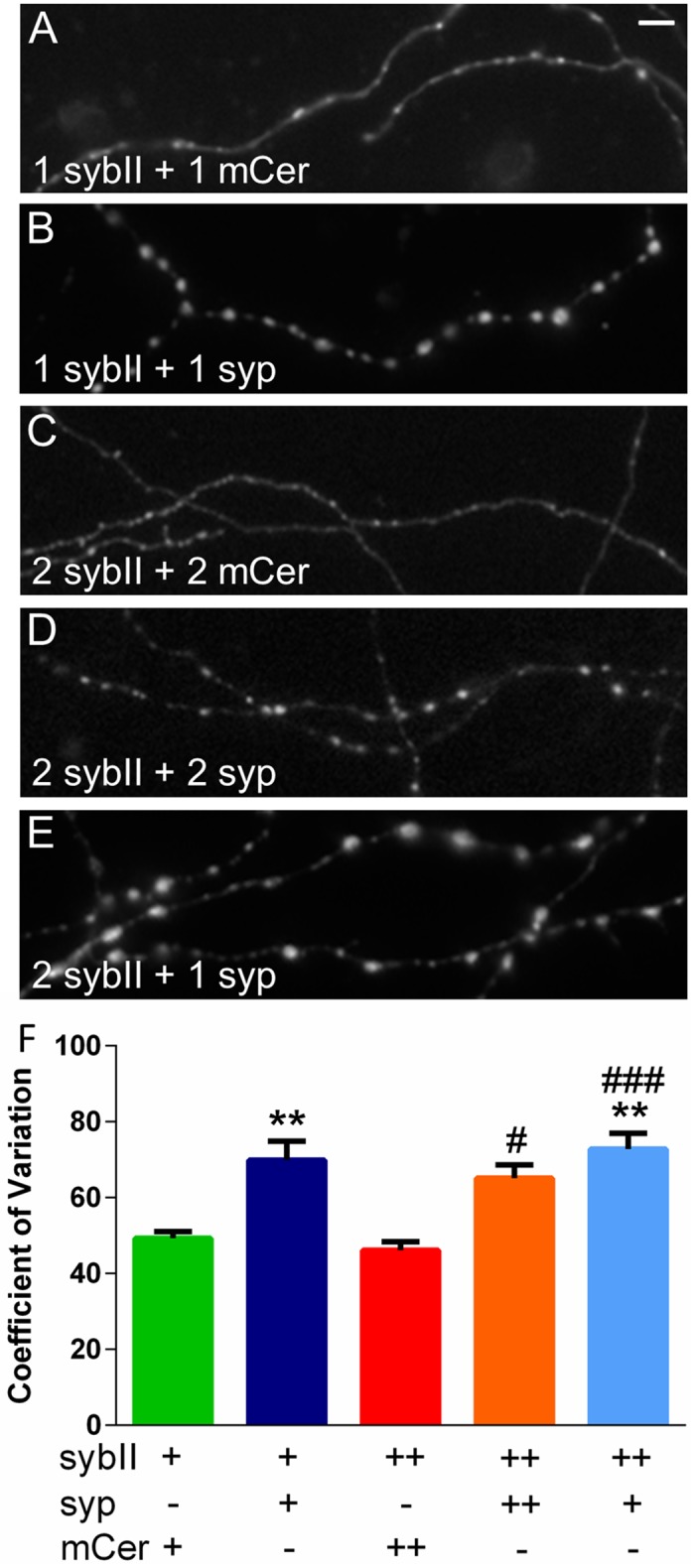
Synaptophysin controls the targeting of sybII to nerve terminals in neurons. Hippocampal neurons were cotransfected with sybII-pHluorin (1 or 2 μg) and either mCer (1 or 2 μg) or syp-mCer (syp, 1 or 2 μg). A-E: representative images of transfected neurons illustrate the differential distribution of sybII-pHluorin in the presence or absence of exogenous synaptophysin. Numbers indicate μg of each construct used. Scale bar represents 15 μm. F: Coefficient of variation of sybII-pHluorin fluorescence intensity along axonal segments, + indicates 1 μg of each construct used, data displayed as mean ± SEM. n = 5–8 (5, 2 μg sybII-pHluorin + 2 μg mCer; 6, 1 μg sybII-pHluorin + 1 μg mCer; 7, 1 μg sybII-pHluorin + 1 μg syp; 8, 2 μg sybII-pHluorin + 1 or 2 μg syp). p < 0.01 (**) against 1 μg sybII-pHluorin + 1 μg mCer; p < 0.05 (#), 0.001 (###) against 2 μg sybII-pHluorin + 2 μg mCer; one-way ANOVA with Holm-Šídák’s multiple-comparison post-test.

The lack of accumulation of exogenous sybII in wild-type nerve terminals transfected in the absence of exogenous synaptophysin could be due to either a mislocalisation of sybII or simply a lack of expression. To delineate between these possibilities, we performed live imaging of neurons to determine the localisation of exogenous sybII-pHluorin (Fig 2). The lumenal pHluorin tag is a pH-sensitive GFP that is quenched in the acidic SV environment but is unquenched in a neutral environment. Neurons were exposed to an alkaline imaging buffer to reveal the expression of sybII-pHluorin in all compartments independent of their pH. SybII-pHluorin was indeed expressed in the absence of exogenous synaptophysin, but was de-enriched from nerve terminals and diffusely localised along the axon (Fig 2A and 2C). In contrast, when co-transfected with synaptophysin, sybII-pHluorin displayed a punctate localisation in neurons (Fig 2B, 2D and 2E), indicating targeting to presynaptic terminals. To quantify this, we measured the CV of sybII-pHluorin fluorescence along axons (Fig 2F). A high CV equates to a punctate localisation, indicating efficient targeting to nerve terminals, while a low CV indicates a diffuse localisation throughout the axon. SybII-pHluorin had a low CV when co-transfected with the mCer empty vector, and a high CV when coexpressed with exogenous synaptophysin. In agreement with the immunofluorescence measurements, 1 μg of transfected synaptophysin DNA was sufficient to fully rescue the punctate localisation and high CV of 2 μg of transfected sybII-pHluorin DNA (Fig 1E and 1F, light-blue bar). Thus, synaptophysin is required for the enrichment of exogenous sybII at nerve terminals, and an approximate 1:2 molar ratio of synaptophysin to sybII is sufficient to direct the latter to the presynapse.

### Synaptophysin expression determines the extent of sybII targeting to SVs

**Fig 3:**
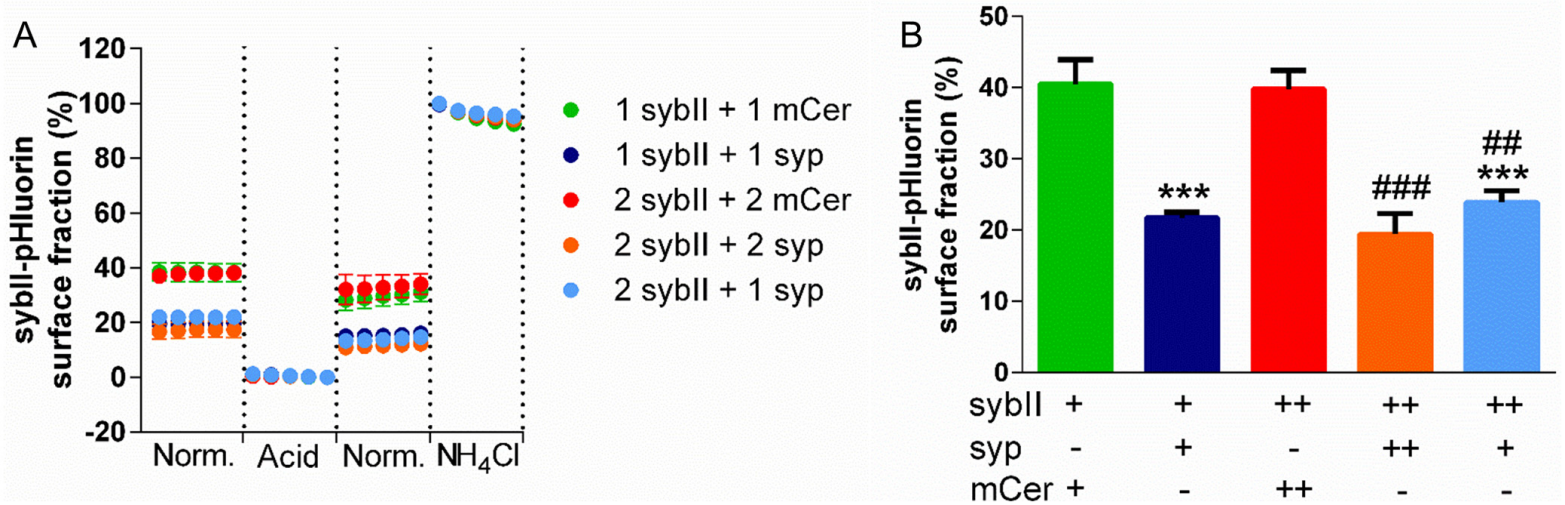
Synaptophysin rescues the plasma membrane levels of sybII-pHluorin. Hippocampal neurons were cotransfected with sybII-pHluorin (1 or 2 μg) and either mCer (1 or 2 μg) or syp-mCer (syp, 1 or 2 μg). n = 5–8 (5, 2 μg sybII-pHluorin + 2 μg mCer; 6, 1 μg sybII-pHluorin + 1 μg mCer; 7, 1 μg sybII-pHluorin + 1 μg syp; 8, 2 μg sybII-pHluorin + 1 or 2 μg syp). A: Average traces (bars indicate SEM) of sybII-pHluorin fluorescence upon exposure to normal (Norm.), acidic or NH_4_Cl buffer, normalised to 100% in alkaline buffer and to 0% in acidic buffer. Numbers indicate μg of each construct used. B: Average surface-localised sybII-pHluorin as a % of total pHluorin fluorescence. Data displayed as mean ± SEM. + indicates 1 μg of each construct used. p <0.001 (***) against 1 μg sybII-pHluorin + 1 μg mCer, p < 0.001 (###) against 2 μg sybII-pHluorin + 2 μg mCer, one-way ANOVA with Holm-Šídák’s multiple-comparison post-test.

In synaptophysin knockout neurons endogenous sybII is mislocalised from nerve terminals, being more diffusely distributed along the axon [12]. This is similar to what is reported here for exogenously expressed sybII that is not cotransfected with synaptophysin. In these synaptophysin null neurons, sybII-pHluorin is trapped at the plasma membrane rather than being efficiently targeted to SVs [12]. We therefore examined whether sybII-pHluorin displays a more prominent plasma membrane distribution in wild-type cells in the absence of additional synaptophysin (Fig 3). As stated above, the pH-sensitive nature of the pHluorin moiety means that in an acidic environment such as the lumen of a SV, its fluorescence is quenched. However when it is localised at the plasma membrane, the pHluorin tag is exposed to the neutral extracellular environment and its fluorescence is unquenched. Therefore the surface fraction of sybII-pHluorin can be estimated by application of an impermeant acidic buffer to quench the plasma membrane-localised reporter, while application of an alkaline buffer reveals the total fluorescent signal.

When sybII-pHluorin was expressed with exogenous synaptophysin, approximately 20% of the reporter was located at the plasma membrane. However, in the absence of exogenous synaptophysin, the amount of sybII-pHluorin at the plasma membrane was doubled (Fig 3A). As observed for the nerve terminal targeting of sybII-pHluorin, 1 μg synaptophysin was sufficient to fully rescue the correct plasma membrane level of 2 μg sybII-pHluorin (Fig 3B).

### A 1:2 synaptophysin/sybII stoichiometry is sufficient to rescue sybII retrieval during endocytosis

**Fig 4:**
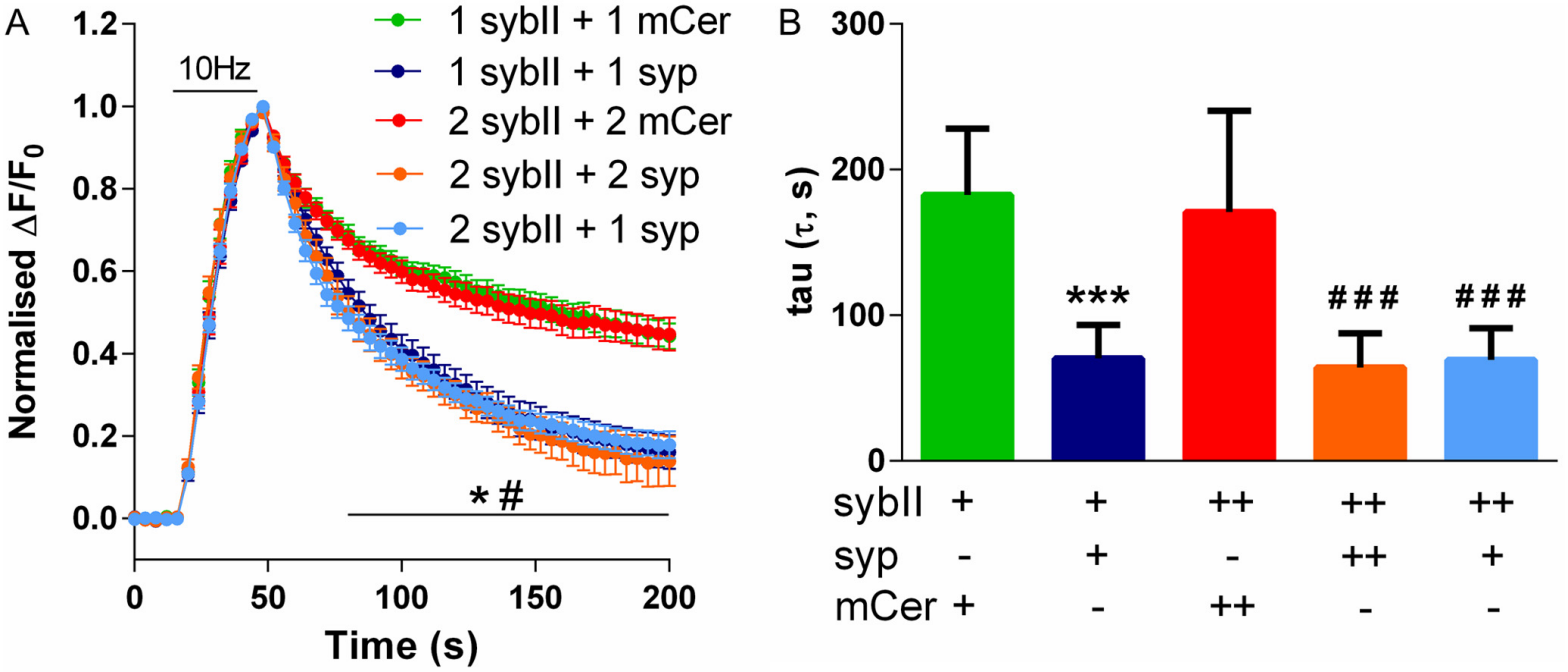
Synaptophysin controls the efficient retrieval of sybII-pHluorin during endocytosis. Hippocampal neurons were cotransfected with sybII-pHluorin (1 or 2 μg) and either mCer (1 or 2 μg) or syp-mCer (syp, 1 or 2 μg), and were stimulated with 300 action potentials at 10 Hz. A: Graph displays the mean ΔF/F0 time course for sybII–pHluorin ± SEM normalised to the peak of stimulation. Numbers indicate μg of each construct used. n = 11 except for 2 μg sybII-pHluorin + 2 μg syp (n = 9) and 1 μg sybII-pHluorin + 1 μg mCer (n = 10). p <0.05 (*) against 1 μg sybII-pHluorin + 1 μg mCer, p < 0.05 (#) against 2 μg sybII-pHluorin + 2 μg mCer over time indicated by bar, two-way ANOVA with Tukey’s multiple-comparison post-test. B: One-phase decay curves were fit to the endocytic portion of the traces to determine tau. Graph displays mean tau (s) ± SEM, n as in A, p <0.001 (***) against 1 μg sybII-pHluorin + 1 μg mCer, p < 0.001 (###) against 2 μg sybII-pHluorin + 2 μg mCer, one-way ANOVA with Holm-Šídák’s multiple-comparison post-test.

The underlying cause of sybII mislocalisation from the nerve terminal and its stranding on the plasma membrane in synaptophysin knockout neurons is due to inefficient retrieval during SV endocytosis [12]. We therefore determined whether this was also the basis of the mislocalisation of exogenous sybII-pHluorin in wild-type neurons lacking exogenous synaptophysin. Transfected neurons were stimulated with a train of 300 action potentials to induce exocytosis, resulting in an increase in sybII-pHluorin fluorescence as SVs fuse with the plasma membrane. The rise time of the sybII-pHluorin fluorescent response was similar in the presence or absence of exogenous synaptophysin (Fig 4A) suggesting that SV fusion was occurring at a similar rate in all conditions. Following the cessation of stimulation, cargo retrieval during compensatory SV endocytosis results in the requenching of pHluorin fluorescence. The downstroke of fluorescence reflects the kinetics of cargo retrieval, since this is rate-limiting when compared to SV reacidification [18,19]. In the absence of exogenous synaptophysin, sybII-pHluorin displays slow retrieval kinetics, indicating inefficient retrieval to SVs (Fig 4A). In the presence of exogenous synaptophysin, sybII-pHluorin retrieval occurs at a faster rate. This was quantified by fitting a one-phase decay curve to the endocytic portion of the traces and measuring the time constant of sybII-pHluorin retrieval (τ, Fig 4B). This confirms that the presence of exogenous synaptophysin is essential for the efficient retrieval of exogenous sybII, and that a 1:2 molecular ratio of synaptophysin to sybII is key to ensure the correct packaging of the latter into newly formed SVs.

## Discussion

Synaptophysin is essential for the efficient packing of sybII into SVs, since in its absence sybII accumulates at the plasma membrane due to perturbed retrieval during endocytosis [12,13]. We extend that finding here to demonstrate that alterations in the molar balance between synaptophysin and sybII results in the dysfunctional retrieval of the latter to SVs. We show that moderate overexpression of sybII (approximately 1.6 fold over endogenous levels) was sufficient to result in stranding of the protein at the plasma membrane due to its perturbed retrieval during endocytosis, even in neurons where synaptophysin is endogenously present. In this system, nerve terminals still maintain at least the normal complement of sybII, but excess sybII is poorly localised to nerve terminals and escapes along the axon. A concomitant expression of synaptophysin in a 1:2 molar ratio with exogenously expressed sybII-pHluorin was sufficient to rescue these defects and highlights the fine balance required between these two key proteins to maintain the correct complement of cargo on SVs.

The fact that a relatively small overexpression of synaptophysin can fully rescue the retrieval of a greater level of sybII parallels the natural stoichiometric balance between synaptophysin (32 copies) and sybII (70 copies) on SVs [1]. However it should be noted that this is an average value, with a larger variability observed when the content of individual SVs are quantified [20]. Moreover, a 1:2 complex between synaptophysin and sybII is also the ratio for the native interaction, which was observed using 3-dimentional negative stain electron microscopy [11,14]. Cholesterol is central to the formation of this complex [14], and depletion of cholesterol from synaptic-like microvesicles results in the de-enrichment of synaptophysin from these vesicles, with a concomitant loss of sybII [21], supporting the concept that a cholesterol-dependent synaptophysin-sybII complex directs sybII retrieval to SVs. The relevance of synaptophysin multimerisation to its functionality remains unclear. The hexameric nature of the central synaptophysin core provides tantalising evidence that synaptophysin may act to cluster sybII dimers. It has been proposed that the purpose of sybII clustering is to ensure efficient SV fusion [14]. However, no deficit in exocytosis rate has yet been identified in synaptophysin knockout cultures [12,22], arguing against such a role. A more likely scenario is that synaptophysin multimerises with sybII dimers on the plasma membrane after SV fusion, simultaneously restricting the entry of the latter into futile cis-SNARE complexes while presenting the SNARE motif of sybII to its adaptor AP180 for retrieval via endocytosis [3]. For this hypothesis to be validated, further work is required to decipher how synaptophysin multimerises, the membranous compartments in which the complex occurs, and the physiological relevance of this multimerisation.

The mistargeting of exogenously expressed sybII has been previously examined. In these studies, overexpressed sybII was stranded on the plasma membrane in either a heterologous cell system [10] or in cultured central neurons [23]. In both instances co-expression of synaptophysin was sufficient to direct sybII to internal structures. It was suggested that synaptophysin acted to target sybII to these internal structures in a manner that was independent of endocytosis [10], however only constitutive endocytosis was examined in this non-neuronal expression system. We clearly demonstrate here that in central neurons, synaptophysin actively facilitates the retrieval of sybII to SVs during compensatory endocytosis in a concentration-dependent manner.

The essential role for synaptophysin in sybII retrieval raises the question as to whether the converse is true for sybII control of synaptophysin retrieval. In support, depletion of sybII in central neurons slows the retrieval of synaptophysin-pHluorin [24], as does depletion of the sybII-specific monomeric adaptor AP180 [19]. However these effects are most likely to be due to a general slowing on SV endocytosis [25,26] rather than specific effects on synaptophysin retrieval itself. Synaptophysin-pHluorin displays decreased plasma membrane stranding compared to sybII-pHluorin when overexpressed in wild-type neurons [19,27]. Thus it is likely that synaptophysin is not dependent on sybII levels for efficient retrieval to SVs, however this requires further examination.

The observation that moderate overexpression of sybII-pHluorin can retard its own retrieval during endocytosis, has implications for the use of this reporter as a surrogate for SV endocytosis. On reflection, the finding that the fine molecular balance between key SV proteins defines cargo retrieval efficiency should not come as a surprise. Indeed it has been acknowledged for many years that when using genetically-encoded pHluorin reporters that only extremely low expressing neurons should be selected for analysis [28]. This study highlights this key experimental point and suggests that when using these reporters to assess SV endocytosis, sybII-pHluorin may not be the most appropriate tool.

Synaptophysin is not the only integral SV protein that interacts with an essential SV cargo. The twelve transmembrane domain protein synaptic vesicle protein 2A (SV2A) binds to synaptotagmin-1 in a phosphorylation-dependent manner [29], and directs its targeting to SVs in a manner reminiscent of synaptophysin-regulated trafficking of sybII [29,30]. Intriguingly, SV2A and synaptotagmin-1 are also present in nerve terminals in an approximately 1:2 ratio [2]. Whether they form a multimeric complex is unknown, however similarly to sybII, synaptotagmin-1 can dimerise [31,32], and can also form ring-like oligomers on lipid membranes [33]. We have recently shown that overexpression of SV2A results in a smaller fraction of surface-localised synaptotagmin-1-pHluorin in wild-type neurons (from approximately 26% in the absence of exogenous SV2A to 7% in the presence of excess SV2A) [29], suggesting that the balance between expression levels of these SV proteins drives the correct targeting of synaptotagmin-1 to SVs in a similar manner to the synaptophysin-sybII complex. It will be important to determine whether common themes define both the synaptotagmin-1-SV2A and sybII-synaptophysin complexes, and whether the ratio of SV2A to synaptotagmin-1 expression drives correct synaptotagmin-1 targeting to SVs in a similar manner to the synaptophysin-sybII interaction.

The revelation that a fine balance between synaptophysin and sybII levels underlies efficient sybII targeting to SVs has implications for the maintenance of efficient neurotransmission throughout an individual’s lifetime. Intriguingly, there is a specific reduction in synaptophysin levels at the earliest stages of Alzheimer’s disease with no change in the levels of other presynaptic proteins such as synaptotagmin-1 or GAP-43 [34]. This would suggest that sybII retrieval to SVs would also be compromised early in AD. We have previously implicated inefficient sybII retrieval in human disease, with intellectual-disability associated variants of synaptophysin all failing to rescue sybII retrieval in synaptophysin null cultures [13]. Small alterations in synaptophysin function or expression levels may therefore result in a decreased fidelity of neurotransmission and synaptic dysfunction.

## Conclusion

In this study we demonstrate that the fine molecular balance between synaptophysin and sybII ensures the correct targeting of sybII to nerve terminals and its efficient retrieval to SVs during endocytosis. Moreover, we show that a 1:2 stoichiometry between synaptophysin and sybII defines the efficient retrieval of sybII, suggesting that this is the threshold copy number required for a functional sybII endocytic complex. This has implications for the maintenance of efficient neurotransmission throughout an individual’s lifetime, and is especially relevant for diseases associated with alterations in synaptophysin expression levels or functionality.

## Abbreviations

SV: synaptic vesicle
sybII: synaptobrevin II
SNAP-25: synaptosomal-associated protein 25kDa
v-SNARE: vesicular soluble NSF attachment protein receptor
mCer: mCerulean
syp-mCer: synaptophysin-mCerulean
MEM: Minimal Essential Medium
CV: coefficient of variation
GFP: green fluorescent protein
SV2A: synaptic vesicle protein 2A
SEM: standard error of the mean
